# Constraints on delivering cell and gene therapies identified during technology appraisal by the National Institute for Health and Care Excellence

**DOI:** 10.1017/S0266462325100391

**Published:** 2025-07-29

**Authors:** Harshini Hariram, Sean P. Gavan

**Affiliations:** 1 Manchester Centre for Health Economics, Division of Population Health, Health Services Research and Primary Care, School of Health Sciences, Faculty of Biology, Medicine, and Health, The University of Manchester, Manchester, UK; 2 Medical Student, Division of Medical Education, School of Medical Sciences, Faculty of Biology, Medicine and Health, The University of Manchester, Manchester, UK

**Keywords:** advanced therapy medicinal product, cell therapy, constraint, gene therapy, implementation

## Abstract

**Objectives:**

Evaluate the extent to which delivery constraints were considered during the health technology assessment (HTA) of cell and gene therapies.

**Methods:**

Constraints on delivering cell and gene therapies were identified from guidance documents by the National Institute for Health and Care Excellence Technology Appraisal and Highly Specialised Technologies streams until October 2024. Inductive coding was performed to identify delivery constraints reported within the guidance documents. A quantitative analysis established the proportion of guidance documents that reported delivery constraints, and the distribution of these constraints across the guidance documents (frequency, mean range).

**Results:**

Sixteen guidance documents for cell and gene therapies were identified. Thirteen guidance documents (81.3 percent of the sample) reported constraints on delivering cell and gene therapies. Thirty-one examples of delivery constraints were reported. The mean number of constraints per guidance document was 1.9 (range: 0–6 constraints). The reported constraints were grouped by six different themes: provider experience (*n* = 8); testing constraints (*n* = 7); geographical constraints (*n* = 5); payment constraints (*n* = 5); maturity of developments in care (*n* = 4); and infrastructure constraints (*n* = 2).

**Conclusion:**

Formal HTA processes are one effective way to identify constraints on delivering cell and gene therapies. Proactive identification of potential delivery constraints will help decision-makers, providers, and manufacturers generate strategies that improve the implementation of cell and gene therapies. Overcoming delivery constraints will strengthen the likelihood of realizing the expected incremental net health benefit of cost-effective cell and gene therapies for patients across a healthcare system.

## Introduction

Effective cell and gene therapies, which offer curative health benefits, are entering routine care across health systems internationally for patients with a range of diseases (1–3). Health technology assessment (HTA) organizations are gaining rapid experience with appraising the value of cell and gene therapies, which has been pivotal to their wider adoption (4–9). However, practical constraints on delivering these treatments remain, which can ultimately affect their expected cost, health benefit, and cost-effectiveness (10;11). There is a growing emphasis on identifying and resolving these constraints, which will help ensure that effective and cost-effective cell and gene therapies are accessible for patients (10). Understanding whether decision-makers, who are responsible for population-level resource allocation decisions, are aware of these constraints will be valuable to help foresee and mitigate similar barriers to adopting future cell and gene therapies during HTA.

Cell and gene therapies are characterized as advanced therapy medicinal products (ATMPs), which are typically administered as a single dose (12). Their use to date has been predominantly for patient populations where no effective alternative treatment is available (6). Cell and gene therapies often have small target patient populations which can result in a limited evidence base at launch (13;14). In conjunction with their relatively high list prices, decision-makers have adopted innovative methods to support value assessment given these data challenges within the framework of cost-effectiveness analysis (e.g., flexible survival extrapolation techniques to explore the duration of treatment effects when faced with complex hazard functions from single-armed trial data) (15–18). As a consequence, example cell and gene therapies deemed to be effective and cost-effective by HTA organizations are being recommended through these processes (5;8). Following these positive recommendations, the responsibility to establish the best arrangements to deliver cell and gene therapies to patients then falls to care providers, system administrators, and health technology manufacturers.

Constraints on the delivery of cell and gene therapies have been known for several years (19–21). Healthcare systems have participated in institutional readiness programs to preempt and resolve these constraints in advance of adopting cell and gene therapies for routine care (22). More recently, practical experience with manufacturing and administering these treatments within standard care settings has led stakeholders to encounter delivery constraints as they arise (23). For example, these constraints can comprise insufficient provider capacity (such as the number of available inpatient beds, the number of specialist nurses trained to manage acute side-effects, or the regional availability of testing centers) and insufficient manufacturing capacity to produce treatments within a timeframe that does not result in patient deterioration (10). Constraints can also arise across sectors, including the need for a robust chain of custody process to ensure the safe transfer of cells between provider and manufacturer institutions (24).

If a cell or gene therapy is deemed to be cost-effective, then delivery constraints will reduce the expected incremental value of care at the population level (10). Delivery constraints will divert patient populations away from the cell or gene therapy and toward the next-best comparator strategy instead. For cell and gene therapies that offer curative intent, the next-best comparator strategies often have low clinical benefit (e.g., end-of-line rescue chemotherapy for cell therapies designed for oncology indications) (25). Fewer eligible patients receiving a cost-effective cell or gene therapy will mean that the expected population incremental net health benefit will not be achieved (10). The value of implementation analysis framework provides a useful method in this context to quantify the net health benefit forgone due to imperfect uptake within a target population (26).

HTA organizations that are responsible for assessing the opportunity cost and health benefits of cell and gene therapies have started to consider whether any practical delivery constraints may exist during their deliberative processes. These considerations will provide a useful source of reference for future cell and gene therapies to anticipate the likely constraints on delivery that may be identified during HTA. Therefore, this study aimed to evaluate the extent to which delivery constraints were considered during HTA of cell and gene therapies.

## Method

This study reports an analysis of constraints on delivering cell and gene therapies that were identified during HTA for the National Institute for Health and Care Excellence (NICE) in England. For the purpose of this study, a delivery constraint was defined as “any factor that impedes or limits the amount of health status produced for a population of patients receiving specified interventions, or policies, provided by the healthcare system” (27). This definition comprises constraints that limit the volume of patients who can receive a cell or gene therapy in routine care settings, and those that affect the incremental cost or health outcomes of care directly (10). Assessments by NICE are a good source of evidence for this information because summaries of committee deliberations are available in the public domain.

### Sample inclusion criteria

The sample for this study comprised all published guidance documents by NICE that provided recommendations for any cell or gene therapy. Guidance documents are the final reports in the public domain that summarize NICE committees’ considerations about the evidence supporting the clinical and cost-effectiveness of the health technology under investigation (28). Cell and gene therapies are currently assessed by NICE through either the Technology Appraisal or Highly Specialised Technologies streams (29). Therefore, guidance documents published by either stream were eligible for inclusion. The dates for inclusion in the sample were from inception until October 2024 (the most recent date available at the time of analysis). There was no restriction on the indicated target patient population. If multiple guidance documents were available for a treatment because they provided recommendations for different target patient populations, then all reports for the different indications were included. If recommendations were updated over time, then the most recent version of the published guidance document was included in the sample. Terminated appraisals were not included in the final sample because a guidance document was not published in these cases. Guidance documents for tissue-engineered products or stem cell therapies without genetic modification were excluded to reduce heterogeneity between the types of treatments included in the analysis.

### Guidance document identification

Relevant guidance documents were identified using two sources. The first source was a recent review of ATMP assessments by NICE, reported by Pinho-Gomes and Cairns (5), which was used to identify published NICE guidance documents for cell and gene therapies until July 2021 (the search date for this study). The second source was a hand search of the NICE website, using the search function to find guidance documents for completed appraisals for cell and gene therapies (date searched: 23 October 2024). Following Gavan et al. (10), the free-text search terms comprised the brand and international nonproprietary names for cell and gene therapies that were approved by the US Food and Drug Administration or the European Medicines Agency (30;31). These sources were used because the relevant regulatory body in the United Kingdom (Medicines and Healthcare Products Regulatory Agency) does not publish an equivalent list of licensed ATMPs. Two authors (HH and SPG) hand-searched the NICE website for candidate guidance documents independently. To decide the final sample, candidate guidance documents were then pooled and read in full by both authors during a meeting to agree on whether a cell or gene therapy was assessed.

### Constraint identification

Two authors (HH and SPG) read the published guidance documents that met the sample inclusion criteria reported in the “Sample inclusion criteria” section in full and independently. The following summary data were extracted from each document: the name of the treatment, the type of treatment, the target population, the date of publication, and the identification number of the guidance document. Constraints on delivering the cell or gene therapy under assessment were identified by qualitative inductive coding independently by two authors (HH and SPG). In this context, a code referred to a word or short phrase that summarized the phenomenon described by excerpts of the guidance document when the text explained a potential constraint on delivery. No restriction was placed on the setting of the constraint (e.g., the care provider or the manufacturer settings). Codes that reflected a similar construct were then grouped together to form themes. These themes were used to define the delivery constraints that were identified in the guidance documents, and the codes provided supporting examples.

### Data analysis

A descriptive analysis reported the summary characteristics of the guidance documents first. The proportion of guidance documents that comprised delivery constraints was then reported by the type of health condition (inherited genetic condition or non-inherited genetic condition). The distribution of the different constraints (themes) across the sample of guidance documents was reported visually. A table summarized the specific delivery constraints that were identified in each guidance document. A quantitative analysis reported the frequency of the different constraints identified across the sample (mean, median, standard deviation, and range). A narrative synthesis explained each identified constraint using supporting examples from the guidance documents.

## Results

The final sample included 16 guidance documents (32–47). Table 1 reports the summary features of these guidance documents, the recommendation by NICE, and whether delivery constraints were identified. The sample comprised guidance documents for eight health technologies to treat inherited genetic conditions (*n* = 5 adeno-associated virus (AAV) gene therapies; *n* = 3 *ex vivo* gene therapies) (32–39). Six treatments for the inherited genetic conditions were recommended by NICE for use in the healthcare system (34–39), and two treatments were recommended with managed access via the Innovative Medicines Fund (32;33). The remaining eight guidance documents were for health technologies to treat cancer indications that were not caused by inherited genetic variation (*n* = 7 chimeric antigen receptor-T (CAR-T) cell therapies; *n* = 1 oncolytic virus) (40–47). Half of these eight treatments (*n* = 4) were recommended for use in the healthcare system via the Cancer Drugs Fund due to uncertainty in the estimates of cost-effectiveness (41;43;45;46), three treatments were recommended for routine commissioning (40;44;47), and one treatment was not recommended because it was deemed not likely to be cost-effective (42).

**Table 1.** Overview of guidance documents

ID	Treatment	Technology	Indication	Published	Recommended	Constraints
Inherited genetic conditions						
TA1003 (32)	Exagamglogene autotemcel (Casgevy®)	*Ex vivo* gene therapy	Transfusion-dependent beta-thalassemia in individuals 12 years and over	September 2024	IMF	Yes
TA989 (33)	Etranacogene dezaparvovec (Hemgenix®)	AAV gene therapy	Moderately severe or severe hemophilia B	July 2024	IMF	No
HST26 (34)	Eladocagene exuparvovec (Upstaza™)	AAV gene therapy	Aromatic L-amino acid decarboxylase deficiency	April 2023	Yes	Yes
HST24 (35)	Onasemnogene abeparvovec (Zolgensma®)	AAV gene therapy	Presymptomatic spinal muscular atrophy	April 2023	Yes	Yes
HST18 (36)	Atidarsagene autotemcel (Libmeldy®)	*Ex vivo* gene therapy	Metachromatic leukodystrophy	March 2022	Yes	Yes
HST15 (37)	Onasemnogene abeparvovec (Zolgensma®)	AAV gene therapy	Spinal muscular atrophy	July 2021	Yes	Yes
HST11 (38)	Voretigene neparvovec (Luxturna®)	AAV gene therapy	Inherited retinal dystrophies caused by *RPE65* gene mutations	October 2019	Yes	Yes
HST7 (39)	Strimvelis®	*Ex vivo* gene therapy	Adenosine deaminase deficiency–severe combined immunodeficiency	February 2018	Yes	Yes
Non-inherited genetic conditions						
TA975 (40)	Tisagenlecleucel (Kymriah®)	CAR-T cell therapy	Relapsed or refractory B-cell acute lymphoblastic leukemia in individuals 25 years and under	May 2024	Yes	Yes
TA895 (41)	Axicabtagene ciloleucel (Yescarta®)	CAR-T cell therapy	Relapsed or refractory diffuse large B-cell lymphoma after first-line chemoimmunotherapy	June 2023	CDF	Yes
TA894 (42)	Axicabtagene ciloleucel (Yescarta®)	CAR-T cell therapy	Relapsed or refractory follicular lymphoma	June 2023	No	Yes
TA893 (43)	Brexucabtagene autoleucel (Tecartus™)	CAR-T cell therapy	Relapsed or refractory B-cell acute lymphoblastic leukemia in individuals 26 years and over	June 2023	CDF	Yes
TA872 (44)	Axicabtagene ciloleucel (Yescarta®)	CAR-T cell therapy	Diffuse large B-cell lymphoma and primary mediastinal large B-cell lymphoma after 2 or more systemic therapies	February 2023	Yes	Yes
TA677 (45)	Brexucabtagene autoleucel (Tecartus™)	CAR-T cell therapy	Relapsed or refractory mantle cell lymphoma	February 2021	CDF	No
TA567 (46)	Tisagenlecleucel (Kymriah®)	CAR-T cell therapy	Relapsed or refractory diffuse large B-cell lymphoma after 2 or more systemic therapies	March 2019	CDF	Yes
TA410 (47)	Talimogene laherparepvec (Imlygic®)	Oncolytic virus	Unresectable metastatic melanoma	September 2016	Yes	No

Abbreviations: AAV, adeno-associated virus; CAR, chimeric antigen receptor; CDF, Cancer Drugs Fund; HST, Highly Specialised Technologies; IMF, Innovative Medicines Fund; TA, Technology Appraisal.

Thirteen guidance documents (81.3 percent of the sample) referred to constraints on the delivery of the cell or gene therapy under assessment (32;34–44;46). Almost all of the eight guidance documents summarizing the committee’s deliberations about gene therapies for inherited genetic conditions included references to delivery constraints (*n* = 7) (32;34–39). By contrast, 75 percent of guidance documents about advanced cell and gene therapy treatments for non-inherited genetic conditions (*n* = 6 guidance documents) included references to constraints on delivery (40–44;46). Table 2 illustrates the distribution of constraint themes that were identified across the sample of guidance documents. Table 3 summarizes the individual delivery constraints that were mentioned within each guidance document.

**Table 2.** Overview of constraints on delivery across the sample of guidance documents

Constraint theme	Published guidance document															
	TA 1003	TA 989	HST 26	HST 24	HST 18	HST 15	HST 11	HST 7	TA 975	TA 895	TA 894	TA 893	TA 872	TA 677	TA 567	TA 410
Provider experience	✔		✔		✔	✔		✔							✔	
Testing constraints				✔	✔	✔	✔	✔								
Geographic constraints				✔	✔	✔		✔		✔						
Payment constraints									✔	✔	✔	✔	✔			
Maturity of developments in care										✔	✔	✔	✔			
Infrastructure constraints						✔									✔	

Abbreviations: HST, Highly Specialised Technologies; TA, Technology Appraisal. Tick denotes that example(s) of that constraint were identified in the corresponding guidance document.

**Table 3.** Constraints on delivery identified within the guidance documents (*n* = 31)

ID	Constraint theme	Example
Inherited genetic conditions (*n* = 19 constraints)		
TA1003 (32)	Provider experience	Staff training
HST26 (34)	Provider experience	Symptom recognition
HST24 (35)	Testing constraints	Genetic testing delay
HST24 (35)	Testing constraints	No newborn screening
HST24 (35)	Geographical constraints	Limited number of provider centers
HST18 (36)	Provider experience	Symptom recognition
HST18 (36)	Provider experience	Awareness of treatment availability
HST18 (36)	Testing constraints	No newborn screening
HST18 (36)	Geographical constraints	Limited number of provider centers
HST15 (35)	Provider experience	Awareness of condition
HST15 (37)	Provider experience	Staff training and education needs
HST15 (37)	Testing constraints	No newborn screening
HST15 (37)	Testing constraints	Test availability
HST15 (37)	Infrastructure constraints	Time to set-up new specialist service
HST15 (37)	Geographical constraints	Travel time to access treatment centres
HST11 (38)	Testing constraints	Genetic testing delay
HST7 (39)	Provider experience	Symptom recognition
HST7 (39)	Testing constraints	No newborn screening
HST7 (39)	Geographical constraints	Limited number of provider centers
Non-inherited genetic conditions (*n* = 12 constraints)		
TA975 (40)	Payment constraint	No tariff for children
TA895 (41)	Payment constraint	No HRG code
TA895 (41)	Maturity of developments in care	Developments in care may reduce toxicity
TA895 (41)	Geographical constraints	Limited number of provider centers
TA894 (42)	Payment constraint	No HRG code
TA894 (42)	Maturity of developments in care	Developments in care may reduce toxicity
TA893 (43)	Payment constraint	No HRG code
TA893 (43)	Maturity of developments in care	Developments in care may reduce toxicity
TA872 (44)	Payment constraint	No HRG code
TA872 (44)	Maturity of developments in care	Developments in care may reduce toxicity
TA567 (46)	Provider experience	Staff training and education needs
TA567 (46)	Infrastructure constraints	Infrastructure to deliver treatment

Abbreviations: HRG, Healthcare Resource Group; HST, Highly Specialised Technologies; TA, Technology Appraisal.

References to 31 examples of delivery constraints were made across the guidance documents. On average, each guidance document contained a mean of 1.9 constraints (standard deviation: 1.6; median: 2; range: 0–6). The constraints were grouped across six different themes: provider experience (*n* = 8); testing constraints (*n* = 7); geographical constraints (*n* = 5); payment constraints (*n* = 5); maturity of developments in care (*n* = 4); and infrastructure constraints (*n* = 2). These constraint themes are next described in detail with supporting examples.

### Provider experience

The experience of healthcare providers in delivering cell and gene therapies was the constraint described most frequently in the guidance documents (*n* = 8). Three guidance documents explained how the diagnosis of inherited genetic conditions may be delayed, given the rarity of the condition, and if presenting symptoms are not specific to the condition eligible for treatment (34;36;39). If providers do not recognize the condition from the presenting symptoms, then the time to administer the gene therapy will be delayed, which may reduce patients’ capacity to benefit. One guidance document explained how earlier diagnosis of spinal muscular atrophy was improving because providers have become more experienced with managing the condition due to a recent increase in available treatments (37). However, providers may also not be aware of the treatments available to manage the condition. For example, one guidance document describes how people with metachromatic leukodystrophy are often not referred to specialist centers by local care providers because they do not think that treatment options are available (36). Three guidance documents described how training was required for the provider workforce to develop skills in administering treatment and managing patients (32;37;46). The responsibilities for delivering this training fell to different stakeholders, including the healthcare system (NHS England) for CAR-T cell therapies (46) and the treatment manufacturer of the AAV gene therapy for spinal muscular atrophy (37).

### Testing constraints

Delivery constraints that were due to testing procedures were reported seven times across the guidance documents for gene therapy health technologies. Four guidance documents explained how newborn screening was not available for the target inherited genetic condition (35–37;39). For example, a newborn screening program would increase the likelihood of diagnosing true cases of presymptomatic spinal muscular atrophy (35) and improve the effectiveness of *ex vivo* gene therapy for adenosine deaminase deficiency–severe combined immunodeficiency (39). Two guidance documents explained how delays to genetic testing for diagnosing the underlying health condition may arise, which will prohibit patients from becoming eligible for treatment (35;38). For example, delays to genetic testing in the healthcare system will likely reduce the effectiveness of gene therapy for presymptomatic spinal muscular atrophy because it will not be possible to treat babies below 6 weeks of age (35). Similarly, the roll-out of the genetic testing program to confirm eligibility for voretigene neparvovec was delayed, which led patient experts to become concerned because access to testing was not universal (38). One guidance document explained that testing antibodies against the adeno-associated vector serotype 9 virus capsid was required before the gene therapy could be administered (37). However, this antibody test was not available in the healthcare system, so the manufacturer of the gene therapy agreed to fund and coordinate this test (37).

### Geographical constraints

Geographical constraints on the delivery of cell and gene therapies were reported five times across the guidance documents. The potential for inequality of access to cell and gene therapies was raised by four guidance documents because of the need to concentrate provider services within a limited number of specialist centers (35–37;41). Patients and their families may need to travel considerable distances to receive treatment, which may not be feasible for some individuals (37). This geographical constraint may reduce the uptake of treatment within the target population and the corresponding net health benefit at the population level. In the context of CAR-T cell therapies, plans to open additional provider centers in more locations will reduce the impact of this constraint on delivery (41). One guidance document explained how the only approved center to manufacture the gene therapy Strimvelis® was in Italy (39). This treatment could only be administered at one Italian hospital, given that its shelf life was six hours (39). Therefore, to overcome this constraint, a bespoke commissioning policy was introduced by the healthcare provider in England, which included travel and accommodation costs for patients and their families (39).

### Payment constraints

Payment constraints were reported five times across the guidance documents. In all cases, these constraints were present in the context of the most recent technology appraisals for CAR-T cell therapies (since 2023) (40–44). For the system in England, healthcare resource group (HRG) codes are used to classify different provider activities when treating people in secondary care (48). Each HRG code has a corresponding tariff representing the monetary unit that providers receive to pay for the underlying activity (48). During four technology appraisals for CAR-T cell therapies, representatives from the healthcare system explained that an HRG code was not available to classify the administration of CAR-T cell therapies (41–44). As a consequence, inconsistencies in the costs incurred between provider centers may arise if different proxy HRG codes are used to classify their CAR-T administration activities. While a tariff is now available to cover CAR-T cell provision (from the decision to have treatment until 100 days after infusion), one guidance report explained how the estimated costs may not be appropriate for children because they may require greater care than adults (40).

### Maturity of developments in care

Delivery constraints that were due to the maturity of developments in care were reported by four guidance documents (41–44). In parallel to the scenario for payment constraints, four guidance documents for CAR-T cell therapies described how the maturity of developments in care may be associated with the degree of toxicity observed in current practice (41–44). The guidance documents explained how clinical developments in care may reduce toxicity from administering CAR-T cell therapy. In practice, these developments may comprise improved administration protocols, ways to predict toxicity in patients, and management strategies to reduce the harm from toxicity.

### Infrastructure constraints

Two guidance documents explained that the necessary infrastructure to provide treatment must be established first before patients can receive cell and gene therapies within routine care (37;46). For example, one earlier guidance document explained how providers must have the necessary infrastructure and safety measures in place first to treat people with CAR-T cell therapies (46). One guidance document for an AAV gene therapy explained how it may take time for the healthcare system to set up a highly specialized service for administering the treatment before patients can benefit (37).

## Discussion

This study found that delivery constraints were reported within the majority of published guidance documents for cell and gene therapies. Committee discussions around the value of gene therapies for inherited genetic conditions were more likely to identify delivery constraints than for non-inherited genetic conditions. Six overarching themes were used to characterize the identified delivery constraints: provider experience, testing constraints, geographical constraints, payment constraints, maturity of developments in care, and infrastructure constraints. A greater understanding of the likely delivery constraints identified during HTA will help decision-makers, care providers, and manufacturers anticipate and overcome common barriers to uptake as more cell and gene therapies begin to enter healthcare systems in the future.

The findings from this study show that formal HTA processes provide a good opportunity to evaluate potential constraints on the delivery of cell and gene therapies before their wider adoption into routine care settings. The example constraints identified by this study align with the Organization Domain of HTA, which is often underreported in HTA documents (49;50). At present, cost-effectiveness is considered independently from any implementation challenges that may arise. However, once economic evaluations depart from assuming perfect implementation of the health technology under assessment, the expected incremental net health benefit of care may decrease (26;51). If constraints on delivering cell and gene therapies are likely to occur, then HTA organizations could adopt value of implementation methods to quantify the expected net health loss due to these constraints (10). This evidence will help to establish the economic importance of delivery constraints and to prioritize resolving those constraints with the greatest economic impact.

The responsibility of resolving constraints on delivering cell and gene therapies will fall to different stakeholders across the healthcare system. In England, ATMPs are commissioned nationally by the highly specialized services portfolio, and provider centers are selected according to clinical expertise and condition prevalence. For this study, healthcare providers within specialist centers who are designated to deliver ATMPs are likely to be responsible for resolving the majority of constraints identified within the guidance documents. For example, providers will need to scale up the infrastructure to deliver CAR-T cell therapies with a reliable supply chain for cell transfer and storage, and deliver sufficient capacity in testing services to confirm genetic diagnoses (19;52;53). There are ongoing efforts internationally to establish ways to overcome these constraints, including the delivery of CAR-T cell therapy in a community setting and gene therapy education programs for low- and middle-income countries (54;55). The guidance documents also indicated how manufacturers can work alongside care providers in supporting strategies to overcome delivery constraints. For example, manufacturers can provide resources to deliver training programs for the healthcare workforce or essential testing services to confirm eligibility for treatment (37). These partnerships will be mutually beneficial for providers and manufacturers if they help to overcome delivery constraints that restrict the number of patients receiving treatment. Future HTA for cell and gene therapies could use the initial scoping phase to work collaboratively with different stakeholders and identify plausible delivery constraints in standard care settings, confirm which parties share the burden of responsibility to resolve these constraints, and establish a timeframe for when the capacity to deliver treatment will be maximized.

The guidance documents also indicated how the type of delivery constraints within a therapeutic class can change over time. For example, early guidance documents for CAR-T cell therapies described how investment in infrastructure and staff training was essential to deliver treatment (46). By contrast, later guidance documents for CAR-T cell therapies explained how payment constraints and the maturity of technology may pose delivery constraints, and no references were made to infrastructure or staff training (40–44). This temporal effect exemplifies that resolving a single constraint may not be sufficient to maximize the value of care because cell and gene therapies are likely to face multiple constraints on delivery (10). Therefore, decision-makers should be mindful that constraints identified for first-in-class cell and gene therapies may not always apply to subsequent treatments within the same class. Early institutional readiness exercises will be essential to preempt the likely evolution of constraints over time and to determine the most valuable way to scale up the delivery infrastructure for handling a greater patient volume for future cell and gene therapies (22;23).

The findings from this analysis of guidance documents were reflected in other published studies. Testing constraints were also identified by Wright et al. (27) in a systematic review of capacity constraints within economic evaluations of precision medicine strategies. For example, a limited supply of trained test providers and testing facilities was found to reduce the uptake of test-and-treatment strategies. A subsequent value of implementation analysis quantified how limited testing capacity for *ALK* mutations reduced the incremental net benefit of crizotinib for people with *EGFR*-negative non-small cell lung cancer (56). A recent systematic review by Gavan et al. (10) found that delivery constraints were reported within published cost-effectiveness analyses of cell and gene therapies. The identified constraints were grouped into four themes: single payment models, long-term affordability, delivery by providers, and manufacturing capability (10). The constraints identified within the guidance documents by this study align mostly with the “delivery by providers” theme (e.g., provider experience, testing constraints, geographical constraints, and infrastructure constraints). By contrast, constraints due to single payment models, long-term affordability, or manufacturing capability were not identified within the guidance documents. This difference may be because alternative payment models, long-term affordability, and manufacturing capabilities are beyond the scope of NICE’s decision-making remit. The findings from the present analysis build on these published studies (which synthesized standalone health economic evaluations) to demonstrate that relevant constraints can also be identified during routine HTA activities that inform resource allocation decisions for healthcare.

One limitation of this study was that the primary data source for the analysis (published guidance documents) may not have reported all examples of delivery constraints that were described during the HTA process. The published guidance documents reflect a summary of the deliberations made by technology appraisal committees rather than a verbatim transcript (28). In addition, potential constraints are not identified or discussed in a systematic way during NICE committee discussions. Therefore, the findings by this study should be interpreted as the lower bound on the number and variety of constraints identified during HTA. A second limitation was that by using published guidance documents by NICE, the findings may be specific to the healthcare setting in England. Healthcare in England is publicly funded and free at the point of use. While the practicalities of delivering cell and gene therapies are similar between public and private insurance-based health systems, alternative delivery constraints may be present in different healthcare settings. A third limitation was that constraints were not reported by whether the health technology was classed as a gene therapy or a cell therapy. This decision was intentional because some cell therapies comprise a gene therapy component, which raises a challenge to distinguish between the two types of treatment clearly. Instead, reporting the constraints according to whether the target population included individuals with an inherited genetic condition or not (Table 3) is likely to be more informative for decision-makers. By doing so, constraints around testing and provider experience were found to be overrepresented for populations with inherited genetic conditions, and payment constraints were found to be overrepresented for populations with non-inherited conditions. Treatments for inherited genetic conditions were clustered around the Highly Specialised Technologies stream because its scope comprises treatments for rare conditions.

Future research could aim to create a taxonomy of likely delivery constraints that can reduce the incremental net health benefit of cost-effective cell and gene therapies. Healthcare decision-makers and care providers could then use this taxonomy as a reference to identify constraints that may affect candidate cell and gene therapies during their decision-making processes. Future research could also undertake value of implementation analyses to quantify the health and resource impacts of delivery constraints for cell and gene therapies (26;51). The cost-effectiveness of alternative implementation strategies can then be assessed to help inform the most valuable way to resolve known delivery constraints. Finally, future research could replicate this analysis using data from published HTA reports in other decision-making jurisdictions. A comparative analysis of the likely delivery constraints between different countries and healthcare settings could then be performed. A global perspective on the presence of delivery constraints can prompt shared learning between jurisdictions and improve the uptake of cost-effective cell and gene therapies internationally.

## Conclusion

Constraints on the delivery of cell and gene therapies were identified during technology appraisals by NICE. These constraints will limit the ability for patients to receive a cell or gene therapy, which has been found to be cost-effective. Early awareness of potential constraints, along with strategies to mitigate their impact, will help to address decision-makers’ concerns about the feasibility of adopting forthcoming cell and gene therapies into routine care settings. Formal HTA processes provide an opportunity to preempt and resolve constraints on delivering cell and gene therapies in routine care settings. Overcoming these delivery constraints will help to ensure that the expected incremental net health benefit of cost-effective cell and gene therapies will be realized for patients across the healthcare system.
